# Potential targeted therapy based on deep insight into the relationship between the pulmonary microbiota and immune regulation in lung fibrosis

**DOI:** 10.3389/fimmu.2023.1032355

**Published:** 2023-01-24

**Authors:** Tao Zhang, Min Zhang, Liqing Yang, Lingyun Gao, Wei Sun

**Affiliations:** ^1^ School of Medicine, Nankai University, Tianjin, China; ^2^ Department of Geriatric Endocrinology, Sichuan Academy of Medical Sciences, Sichuan Provincial People's Hospital, Chengdu, China; ^3^ Department of Respiratory and Critical Care Medicine, Sichuan Provincial People's Hospital, Chengdu, China; ^4^ Sichuan Provincial People's Hospital, Sichuan Academy of Medical Sciences, Chengdu, China; ^5^ Medical College, University of Electronic Science and Technology, Chengdu, China; ^6^ Guanghan People's Hospital, Guanghan, China

**Keywords:** lung microbiota, immune regulation, microecology, lung fibrosis, IPF – idiopathic pulmonary fibrosis

## Abstract

Pulmonary fibrosis is an irreversible disease, and its mechanism is unclear. The lung is a vital organ connecting the respiratory tract and the outside world. The changes in lung microbiota affect the progress of lung fibrosis. The latest research showed that lung microbiota differs in healthy people, including idiopathic pulmonary fibrosis (IPF) and acute exacerbation-idiopathic pulmonary fibrosis (AE-IPF). How to regulate the lung microbiota and whether the potential regulatory mechanism can become a necessary targeted treatment of IPF are unclear. Some studies showed that immune response and lung microbiota balance and maintain lung homeostasis. However, unbalanced lung homeostasis stimulates the immune response. The subsequent biological effects are closely related to lung fibrosis. Core fucosylation (CF), a significant protein functional modification, affects the lung microbiota. CF regulates immune protein modifications by regulating key inflammatory factors and signaling pathways generated after immune response. The treatment of immune regulation, such as antibiotic treatment, vitamin D supplementation, and exosome micro-RNAs, has achieved an initial effect in clearing the inflammatory storm induced by an immune response. Based on the above, the highlight of this review is clarifying the relationship between pulmonary microbiota and immune regulation and identifying the correlation between the two, the impact on pulmonary fibrosis, and potential therapeutic targets.

## 1 Introduction

With the development of the study on the pathological mechanism of interstitial lung disease (ILD), basic medical research is paying more and more attention to the pulmonary microbiota and immune regulation mechanism of lung fibrosis. The influence of immune regulation on the lung microbiota may be the core event of the progression of pulmonary fibrosis (1, 2). Previous research has found that abnormal intestinal microbiotas destroy the immune barrier and participate in immune-related diseases in terms of different strains, the proportion of flora, and opportunistic pathogenic bacteria. The occurrence and development of human diseases are closely related. The lungs and intestines are connected with the external environment, and there are colonized microbiota and opportunistic pathogenic microorganisms in the microbiota. Bronchoalveolar lavage fluid (BALF) metagenomics has confirmed different bacterial structures in other states of ILD. Meanwhile, various pathogenic bacteria seriously affect the progress and prognosis of lung fibrosis (3, 4). Previous studies have shown that intestinal microbiota and immune regulation are inseparable. A stable microbiota is vital to maintaining an immune balance. Once the lung microbiota is unbalanced, it will result in aberrant immunological signal transduction. On the one hand, the above biological effects will cause the reproduction of pathogenic bacteria in a vicious cycle, further damage the pulmonary microbiota, and participate in the occurrence and development of lung fibrosis (5, 6). On the other hand, the unbalanced microbiota will cause the antigen-presenting cell (mainly macrophage), B cell, T cell, and natural killer cell to participate in pathological biological effects. The cells mentioned above are immune executors, further activating the signal pathway related to pulmonary fibrosis, causing the polarization state of macrophages in the lung stroma to change and the transformation of intrinsic fibroblasts into myofibroblasts, aggravating the progress of IPF. In the process of protein participating in immune regulatory response, more than 50% of the proteins are glycoproteins These proteins are involved in lung fibrosis while performing the body’s immune regulation. The literature has reported that core fucosylation (CF), a critical functional glycoprotein modification, regulates pathological immunity. And CF becomes a crucial link in myofibroblast accumulation and extracellular matrix deposition (ECM) in the lung stroma by regulating the activation of multiple signal pathways (7–9). Because of the above, the current means to remodel the lung microbiota includes antibiotic therapy, Vitamin D supplementation, and micro-RNA. To a certain extent, although some patients with AE-IPF benefit, the lesion of IPF still cannot be reversed. Therefore, it is reasonable to speculate that the future modification of CF between immune regulation and lung microbiota may become a possible new strategy for treating IPF. In this review, we review three points. 1. The difference of lung microbiota in different pathophysiological states of pulmonary fibrosis. 2. The immune response to abnormal lung microbiota. 3. Based on immune regulation after microecological imbalance, current therapeutic direction.

## 2 Pulmonary microbiota

### 2.1 Pulmonary microbiota in healthy people

The limitations of traditional pathogenic microbial techniques, previous studies thought that patients’’ lungs were sterile. However, with the development of high-throughput sequencing technology, more and more researchers found that the pulmonary microbiota consisted of various flora distributions in pulmonary microbiota. In ordinary healthy people, lung microbiota originate from the upper respiratory tract due to the continuity of airway anatomy (10). As usual, lung colonization is lipophilic microorganisms, including propionibacterium, Staphylococcus, and Corynebacterium (1). On the other hand, a study reported that lung microbiota was not entirely similar to the upper respiratory tract. Dickson et al. found Tropheryma Whipple, the bacteria not found in the upper respiratory tract (2). Therefore, it breaks the traditional opinion that the upper respiratory tract is the only source of microorganisms in the lower respiratory tract. Besides, we found that respiratory tract microbes may be various in different populations, such as newborns (11). Biological colonization also has specific differences because of other delivery methods (12). In natural childbirth, the pulmonary mucosa microbial species in the skin, oral, pharynx, and intestinal tract were similar to those in the mother’s vaginal microorganisms (13). However, the microbial community in a cesarean fetus was more identical to the organisms in its mother’ s skin and environment (14, 15). To be summarized, the microbiotas in the healthy lung have some variations in different anatomical localization.

### 2.2 Pulmonary microbiota in IPF

Candida, Neisseria, Actinomycetes, and other series were first found in BALF and extracted from 17 patients with stable idiopathic pulmonary fibrosis (IPF). Another Japanese study proved that the most prevalent lung phyla were Firmicutes, Proteobacteria, and Bacteroidetes. Meanwhile, decreased microbial diversity was in 8 AE-IPF patients in the deterioration group. Additionally, decreased abundance of Firmicutes, Streptococcaceae, and Veillonellaceae were significantly associated with the progression of ILD (16). In 2019, a study reported that the lung microbiota of IPF in bleomycin-induced mice and their bacterial abundance was higher. The Haemophilus, Candida, Neisseria, and Weberia of IPF were more abundant *via* the dysregulated microbiota. Besides, these dysregulated microorganisms could stimulate persistent alveolar damage and cause the progression of ILD. In turn, interleukin-17B (IL-17B) production demonstrates that dysregulated lung commensal bacteria drive IL-17B production to promote pulmonary inflammation and fibrosis through their outer membrane vesicles (17).

On the other hand, some studies found lung microbiota related to gene polymorphism. For instance, mucin gene promoter polymorphism of rs35705950 increases the survival rate of IPF. Furthermore, the gene polymorphism could enhance the immune tolerance of pathological microbiotas and regulate the protein post-translational modification (18). Something deserves attention, and there are also significant differences in oral bacteria among IPF patients. This phenomenon indicates microbial selection in the lower respiratory tract is in the process of pulmonary fibrosis. It is also unclear whether the oral pathogenic bacteria is close to the pulmonary microbiota (19). Therefore, a larger sample size is needed to confirm the change in bacterial abundance and the selection of dominant flora in pulmonary microbiota in IPF.

### 2.3 Pulmonary microbiota in AE-IPF

AE-IPF refers to the occurrence of new diffuse alveolar injury, which leads to the progressive aggravation of restrictive ventilation dysfunction. We cannot avoid the poor prognosis of IPF, but we should actively look for the causes of AE-IPF (20, 21). The mechanism of AE is unclear, but it is clear that infection is involved in lung fibrosis. Pathogenic microorganisms participate in AE-IPF. These are also essential members of the lung microbiota. For instance, staphylococcal could cause acute alveolar damage. It ruins the biological characteristics of pulmonary microstructure. Aspergillus could result in non-necrotic granulomatous lesions.

**Figure 1:**
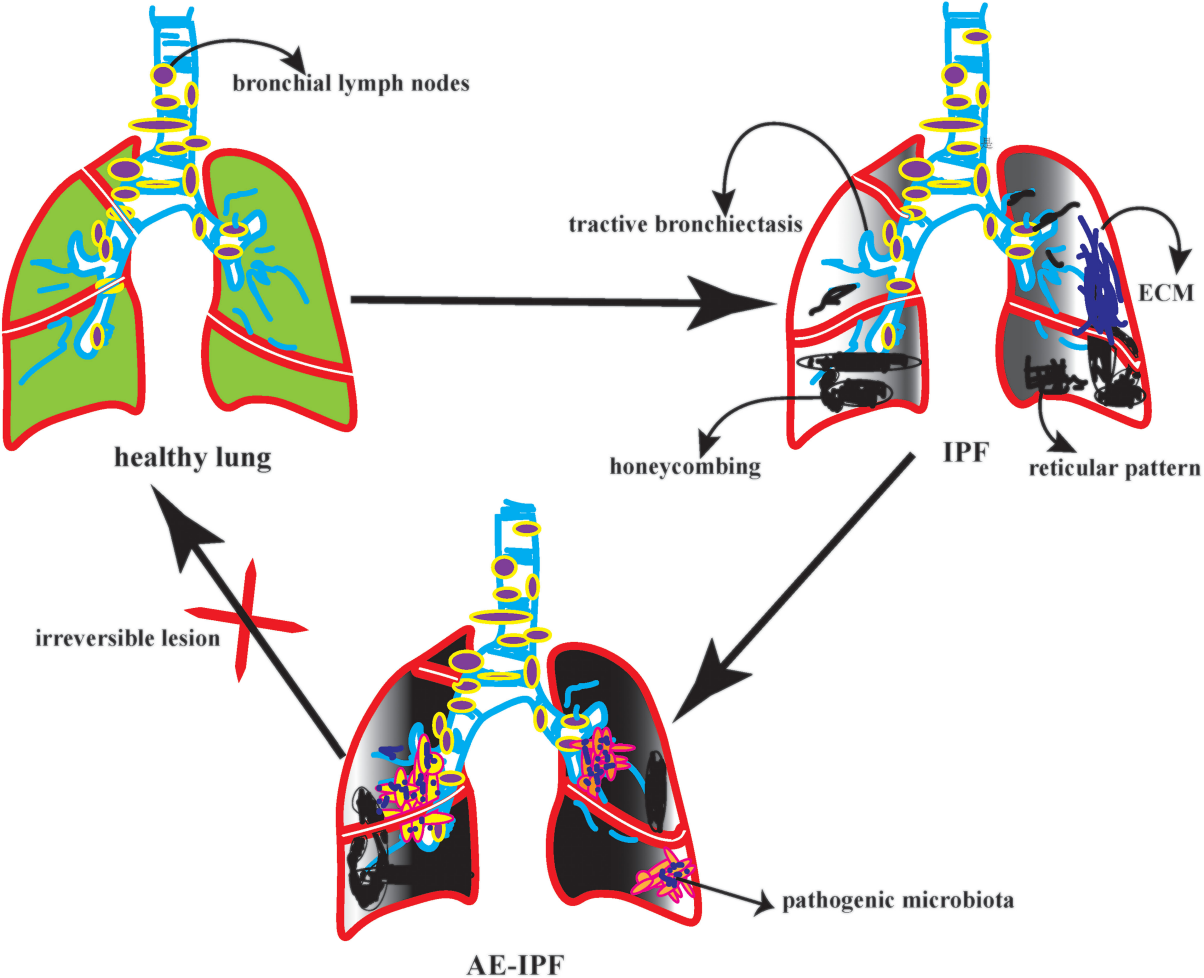
The picture depicted the different pulmonary microbiota in various state of ILD.

On the other hand, it accelerates the inflammatory cytokines recruitment in the exudative stage. A prospective study showed that among 65 patients with IPF, FVC decreased by more than 10% in 22 patients. AE-IPF was rated as the diagnosis of 22 patients. After observing the bacterial load, structure, and composition in BALF patients with AE-IPF. It found that the bacterial load of AE-IPF was much higher than that of IPF; however, there was no significant difference in bacterial structure and composition (22). The reduction of bacterial diversity could activate the immune regulation of lung microbiota and indirectly lead to new diffuse lung injury (23). Therefore, the increase in bacterial load is closely related to acute progression and high mortality of AE-IPF (24, 25). In a case-control study in Korea, 18 patients with AE-IPF had a higher bacterial load in BALF than 14 patients with stable IPF. However, at the operational taxon level of BALF, Campylobacter and P. Elongatum increased significantly in AE-IPF, while P. veronicas decreased significantly. The results of this study are inconsistent with the previous literature (26). Therefore, we further reasoned that this result might be due to the following reasons: First, the sample size is too small. The second reason, some IPF patients in this study received anti-infection treatment. The third reason, the study lacked oropharyngeal specimens as controls. To be summarized, in the future acquisition of clinical BALF, we need to seriously consider the bronchial sampling route, the target lobe of lavage, and the loss of negative control, which will indirectly affect the highly sensitive flora sequencing. In particular, AE-IPF, a disease with many confounding factors, high heterogeneity, and unknown underlying disorders, should be interpreted with more caution. (**Figure 1**)

## 3 Immune response to abnormal lung microbiota

### 3.1 Innate immunity response to abnormal microbiota

In a broad sense, the change in microbial abundance refers to the evolution of microbial structure, proportion, and colony (27). On the level of chivalry, the change of microbial abundance mainly consists of the reduced dominant flora, inverted microbial ratio, opportunistic pathogenic bacteria multiplying, and unbalanced microbiota (28). Innate immunity is the immunological guardian because of the abnormal microbial abundance change (29). In the first step, the macrophage was the significant mediated cell in natural immunological response (30). For instance, a previous study reported that the function of macrophages could be ruined when aspergillus is pathogenic (31).

Furthermore, the abnormally aspergillus proliferation could release some inflammatory medium, including matrix metalloproteinases-3(MMP-3) and matrix metalloproteinases-9(MMP-9), which represses mitochondrial respiration and oxidative stress and produce overloaded oxygen free radical (32, 33). Thirdly, MMP-9 is related to the upregulated TGF-β pathway in fibroblasts, which activates this pathological fibrosis (34). Finally, it ruins antigen-presenting function and formats the pathological foundation of lung fibrosis (35). On the other hand, macrophage polarization also participates a critical role in innate immunity in the process of pulmonary fibrosis (36). For instance, fungi other than aspergilli, such as schizophyllum, could induce allergic bronchopulmonary mycosis (ABPM). And it stimulated the pre-stage lesion of pulmonary fibrosis (37). Further consideration, the biological characteristics of fungi could destroy the structure of the dominant flora in the lung and then produce some inflammatory chemokines (such as CCL17, IL-13, and M-CSF) (38), which accelerated the pathological injury of macrophage polarization and recruited the stronger inflammatory storm in the involved lung (39). Therefore, microbial abundance change could take some pathological effect on the innate immunological response, mainly influencing antigen presenting process and macrophage polarization (40).

### 3.2 Specific immunity response to abnormal lung microbiota

As usual, the specific immunity could divide into cellular and humoral. However, the two others are closely linked and share some pathological signal transduction. The abnormal pulmonary microbiota often could influence the B cell function and T_H_-B cell interaction (3). For instance, a previous study reported that the over-loaded Haemophilus would increase the recruitment chemotaxis of neutrophils. And further recruit more chemokines to exudative lesions in the early stage of lung fibrosis (41). Recently, a study found that the release of neutrophil elastase is an essential link in cystic pulmonary fibrosis. Because neutrophil elastase could destroy the pulmonary capillary barrier and activate the B cell immune response (4, 42), the abnormal pulmonary microbiota can lead to Staphylococcus becoming an opportunistic pathogen (43). Staphylococcal protein A (SPA) is a surface protein of staphylococcal, which combines with mucin of the cell wall. It binds to the Fc segment of IgG in humans and mammals (44). SPA affects innate cellular immunity because it has anti-phagocytosis. SPA also hinders the specific binding of antigens and antibodies because SPA is the carrier of Staphylococcus. Because SPA indirectly weakens the neutralization and clearance of the antibody to the particular antigen (45, 46). On the other side, Staphyolysin could constrict small blood vessels, cause local ischemia and necrosis, and cause smooth muscle spasms (47). For instance, α-hemolysin is an exotoxin, which can result in the collapse and necrosis of capillaries. It is the pathological foundation of angiogenesis in lung fibrosis (48). Thereby, the over-loaded Haemophilus or the opportunistic pathogenic Staphylococcus may play a critical role in affecting cellular immunity. Antibody-dependent cell-mediated cytotoxicity (ADCC) involves many immunological regulation (49). ADCC is also an integral approach to cellular apoptosis. And participate in the alveolitis of the denaturation phase in lung fibrosis (50). First is the inflammatory cytokines released by the M2 and NK cells. However, M2 cells are a large family, and different subtypes of M2 affect immune response (51). Previous studies reported that fungi or aspergillus could influence the differentiation of macrophages.

Then, unbalanced microbiota leads to the auto-immune disorder of lung fibrosis. As the secreting IL-4, IL-10, and PDGFA (52). Finally, it results in the over-killing effect of ADCC, which induces the beginning of lung fibrosis. On the other hand, the function of NK cells depends on the regulation of chemokines, and the appropriate level of chemokines may alleviate diffuse alveolar damage (53). However, an unbalanced pulmonary microbiota is the ideal culture medium for chemokines, such as MMP-13, IL-10, and CCL22 (54, 55). In this case, the over-expression of chemokines could enhance the ADCC and promote the persistent injury of the pulmonary lesion in lung fibrosis. To be summarized, the unbalanced flora structure or pathogenic colony might be a significant step for adaptive immune response in lung fibrosis.

## 4 Immune regulation

### 4.1 CF as the primary immune regulation

Firstly, fucosylation is the modification of protein post-translation and includes two aspects. 0-glycosyl type took the hydroxyl groups of serine, threonine, hydroxylysine, and hydroxyproline as the connecting points. With the phenol amino group of asparthalamide and the N-terminal amino acid α - Amino and lysine or arginine ω - The amino group is the connecting point, forming the N-glycosyl type. In the second place, N-glycan fucosylation is equipped with stable physicochemical properties. And might be considered the ideal targeted direction of IPF therapy. Thirdly, CF is the targeted measure to regulate many immunological responses in protein post-translation. CF is catalyzed by fucosyltransferase 8 (Fut8). CF is the significant fucosylation pattern on the surface of glycoproteins (56). In detail, Fut8 transfers the guanosine diphosphate-fucose (GDP-Fucose) to the sixth carbon atom on the N-acetylglucosamine (GlcNAc) of the N-glycan, forming an α-1, 6-glycosidic bond which called as CF (57). It is reasonable to propose that CF could affect the glycosidic linkage flexibility and conformation of proteins, resulting in modification of protein interactions or assembly. CF participates in many immunological responses, especially in senescent alveolar epithelial cells (AECs) (58). The senescent AECs have the potential to differentiate the myofibroblast, which is the initiator of extracellular matrix accumulation, consequently, the formation of the basis of pulmonary fibrosis (59). Previous studies reported that Fut8 gene knockout dramatically impacts the function of glycoproteins, which exerts an enormous part on immune responses. To be summarized, antigen-presenting cell expression (mainly macrophage), cell transduction, and antibody-dependent cell-mediated cytotoxicity (ADCC) were closely related to CF (60, 61).

### 4.2 CF regulates macrophage polarization

**Figure 2:**
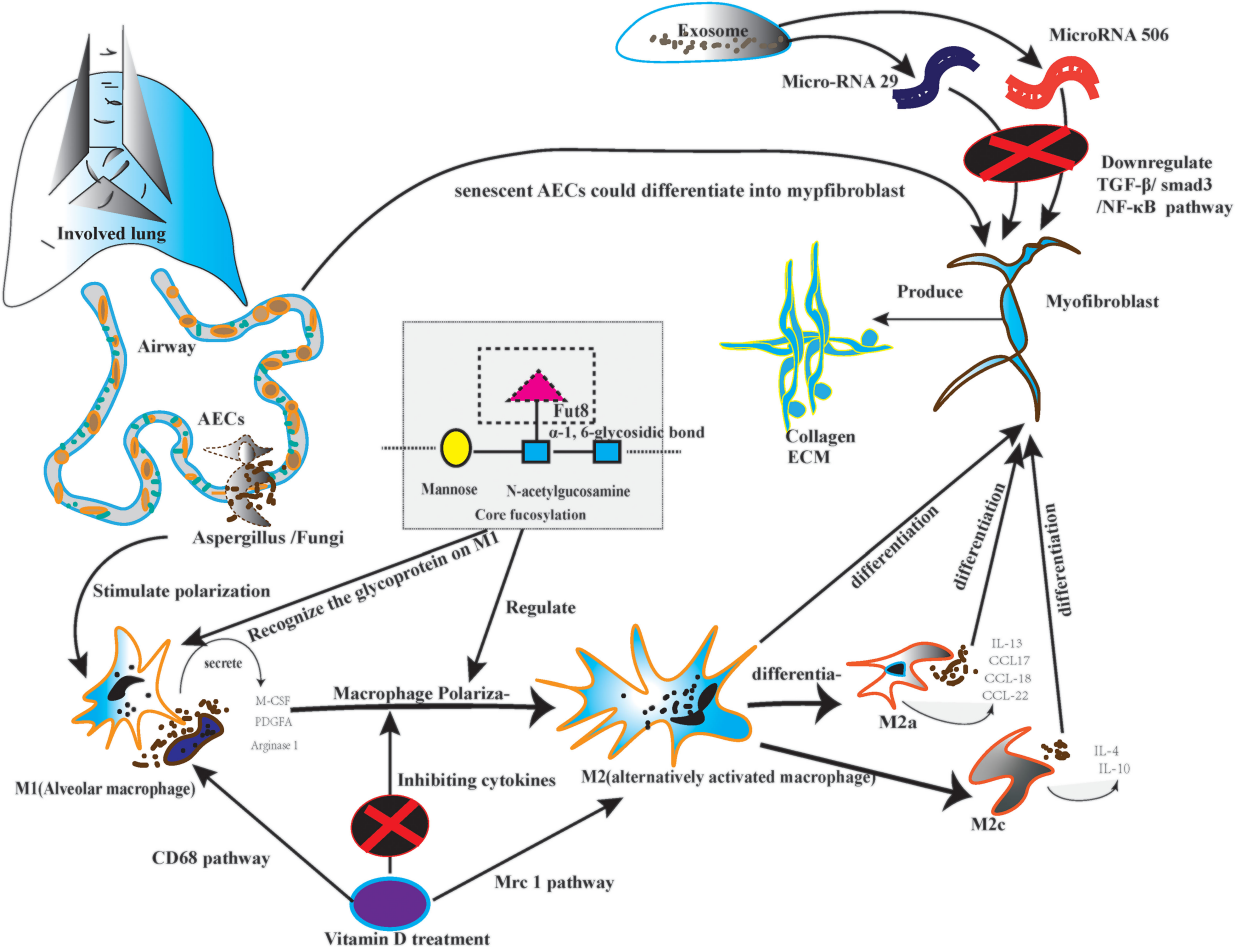
The picture depicted the core fucosylation regulating macrophage polarization on lung fibrosis pathology.

The macrophage and dendritic cells are the primary antigen-presenting cell; the antigen-presenting cell plays a significant role in immunological response. On the other hand, the CF regulates the macrophage, particularly macrophage polarization (62). Firstly, the macrophages could be classified into classically activated macrophages (M1) and alternatively activated macrophages (M2) (63). M2 could differentiate into four subtypes, including M2a, M2b, M2c, and M2d. Every subtype of M2 plays a role in lung fibrosis (64). Secondly, as the post-translational glycoprotein modification, CF affects macrophage polarization, which is the core step of lung fibrosis, especially on the N-glycans of the surface glycoproteins (65). Meanwhile, macrophage polarization needs glycoproteins to recognize signal information (66). Monocyte-derived macrophages secrete macrophage colony-stimulating factor (M-CSF) in an autocrine manner. Monocyte-derived macrophages also produce Platelet-derived growth factor subunit A (PDGFA) and matrix metallopeptidase 13 (MMP13). All of them could stimulate lung fibrosis (5, 6). Thirdly, alveolar and monocyte-derived macrophages can be polarized into the M2 phenotype (67). M2 macrophages produce tissue growth factor-β1 (TGF-β1), inducing the differentiation of fibroblasts into myofibroblasts (68). Fourth, M2a cells can promote the Production of interleukin-13 (IL-13) and several other chemokines, such as C-C motif chemokine ligand 17 (CCL17), CCL18, and CCL22, which is the foundation of pulmonary inflammatory injury (69). M2a also execute clearing by phagocytosis; they actively participate in extracellular matrix (ECM) remodeling and angiogenesis. All the steps are the premise of pulmonary fibrosis (70). Conversely, the M2c could initiate the overexpression of IL-4 and IL-10 under persistent inflammatory factor stimulation (71). Finally, M2 can also depolarize to M0 macrophages or exhibit the opposite phenotypes by repolarizing. Macrophage polarization depends on the cytokines in the specific pulmonary microbiota (72). Because different lung microbiota possess various bacterial loads, structures, and tolerance. Diverse microbiota may cause various immunological cytokine secretions. Therefore, CF may have the potential approach to solve the abnormal repair of macrophage polarization in certain lung microbiota. (**Figure 2**)

### 4.3 CF regulates the T_H_-B cell interaction

**Figure 3:**
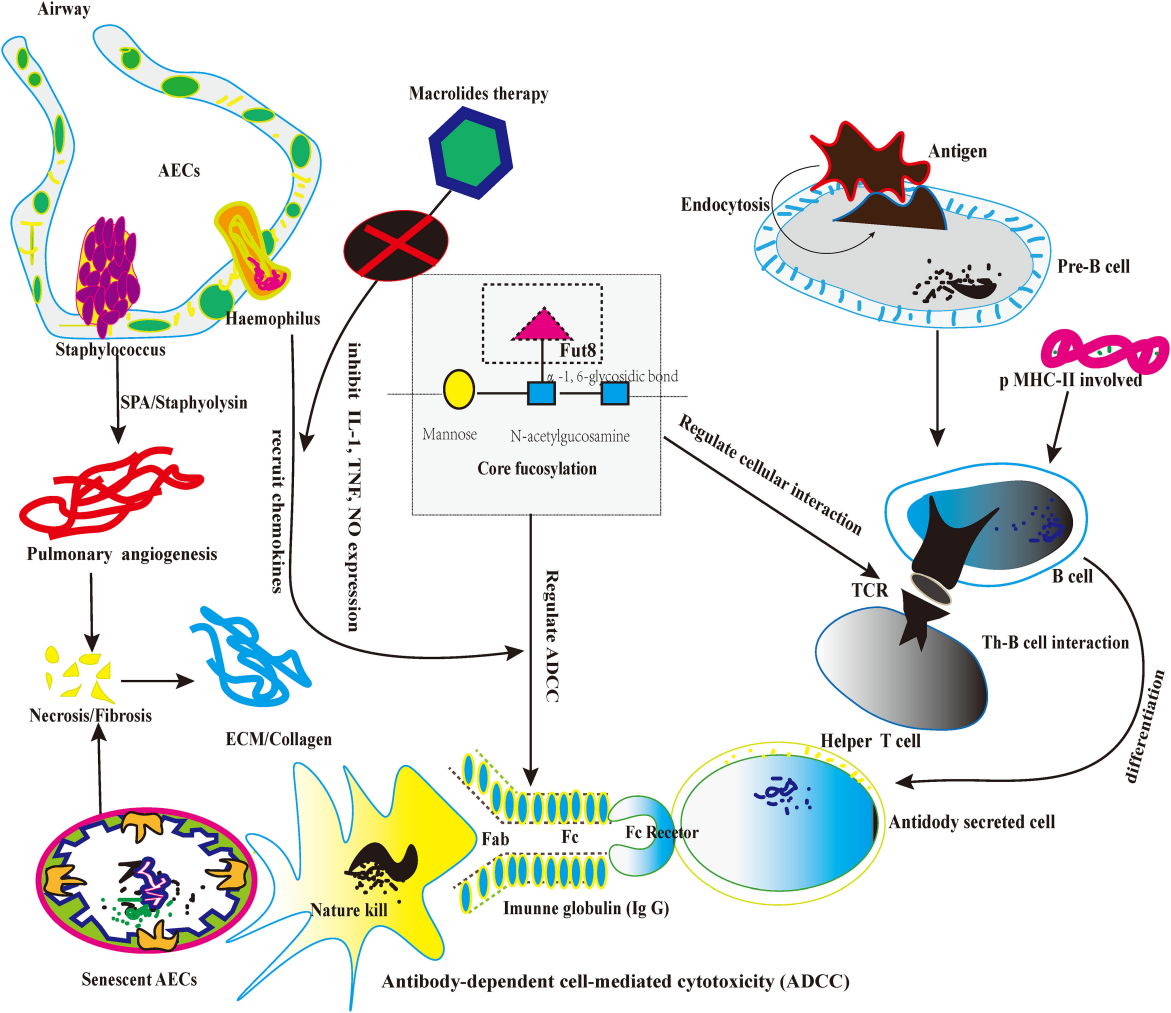
The picture depicts the core fucosylation regulates ADCC and lymphocyte information transduction about lung fibrosis.

Firstly, CF regulates the pre-B cell receptor (pre-BCRs). The functional pre-BCR complex consists of a constant region of the heavy chain (UHC), immunoglobulin (Ig), and surrogate light chain (SLC) (73). Loss of CF could influence the pre-BCR assembly and reduce the binding affinity between the targeted antigen (Ags) and pre-BCR (74). Secondly, pre-B cells differentiate into mature B-cells upon B cell activation. B-cell recognizes both soluble and membrane-associated antigens (Ags) by BCRs with the help of antigen-presenting cells (7), and B-cells immediately respond to external stimulation (8). After recognizing Ags, BCRs on B-cells could trigger signal transduction that eventually induced B-cell activation and antibody (Abs) production. Loss of CF reduced the lipid raft formation of B cells and alleviated the overexpression of BCRs recognition (9). Thirdly, B cells could process and present the Ags peptide with the help of major histocompatibility complex class II (MHC-II). Then, the peptide-loaded MHC-II complex (pMHC-II) on B cells could be recognized by Ag-specific armed T-help cell (T_H_ cell) *via* T cell receptor (TCR) (75). Lastly, During the T_H_-B interaction, activated T_H_ cells produce cytokines for the B lymphocyte’ s clonal expansion and differentiate into Ab-secreting cells. Therefore, the appropriate T_H_-B exchange would take a protective effect (76). However, excessive T_H_-B exchange may take pathological effects, especially the lung fibrosis possesses persistent severe inflammatory response storm (SIRS) (77). For instance, CF could promote the recruitment of TCR to the synapse and enhance TCR internalization, enhancing the immune response and boosting apoptosis, inducing the necrosis of AECs (78). As we know, the senescent AECs are the origins of diffused alveolitis damage and the foundation of lung fibrosis. To be summarized, the knockdown of the Fut8 may be the potential targeted option to solve the hyperactivity of T_H_-B interaction because Fut8 could down-regulate CF expression (79, 80). (**Figure 3**)

### 4.4 CF on regulating the induction of ADCC

Firstly, CF occurs typically in human immunoglobulin G (IgGs) and plays a significant role in IgG function. As we know, IgG participates in the ADCC and mediates apoptosis in cellular death (81). Secondly, a previous study proved that each IgG molecule has a highly conserved N-glycan at Asparagine (Asn) in the heavy chain2/heavy chain 3 (CH2/CH3) domain. “““““. The highly conserved N-glycan plays a crucial role in sustaining the conformation of the Fc domain of IgGs (82). Thirdly, multiple sugar chain moieties extend from Fc domains toward each other into the IgG interchain region and stabilize the IgG framework (83). On the other hand, CF N-glycans attached to the Fc region are a critical determinant of ADCC, as the deletion of core fucose from the Fc region enhances its binding affinity to the fragment crystallizable receptor” (FcgRs) and significantly improves ADCC (84). The de-fucosylated IgG-Fc domain enhanced the induction of ADCC about 50~100-fold (85, 86). Therefore, future studies could take CF as the artificial intervention of the ADCC approach (87). Furthermore, de-fucosylated modification may alleviate the abnormal apoptosis by natural killer cells (NK cells), which indirectly relieves the aberrant immunological response of fibrotic pathology (88). In future studies, regulating the balance between CF and de-core fucosylation in lung fibrosis might be a new potential targeted therapy. (**Figure 3**)

## 5 Potential targeted therapy based on the relationship between lung microbiota and immune regulation

### 5.1 Antibiotic therapy

It remains unclear whether bacterial superinfection ILD directly triggers fibrosis in individuals with underlying ILD. However, the clinical report demonstrated that infections accelerate the deterioration of lung fibrosis, and antibiotic treatment reduces mortality in these patients (89, 90). Something deserves our attention. A previous study found that macrolides, such as erythromycin, clarithromycin, and azithromycin, are antibiotic compounds. They are effective against Gram-positive and Gram-negative bacteria. Including streptococci, Haemophilus, staphylococci, mycoplasma, mycobacteria, and chlamydia (91). Apart from their established antibacterial effects, there is growing evidence for an immune-regulating effect of macrolides (92). Firstly, the dual effect of macrolides on bacteria and host immune cells (93). Macrolides inhibit bacterial protein translation by interfering with the ribosome subunit. They reduce biofilm production and bacterial adherence (94). Secondly, an immunomodulatory effect is achieved *via* lowering the Production of inflammatory cytokines, such as tumor necrosis factor (TNF), interleukin-1 (IL-1), and nitric oxide (NO) (95, 96).

Conversely, macrolides could increase IL-10 as an anti-inflammatory cytokine (97). IL-10 is one of the most important anti-inflammatory cytokines produced by T or B regulatory cells (16). IL-10 is central in protecting host tissue during infection by inhibiting the synthesis of interferon-gamma (IFN-γ) synthesis by both T cells and NK cells (98). Therefore, the macrolides might become a potential targeted option. When abnormal pulmonary microbiota mainly includes Gram-positive and Gram-negative bacteria. However, when fungi cause microecological lung disorder, antifungal treatment is still controversial because it lacks the support of basic experiments.

### 5.2 Vitamin D supplementation

The latest study reported that Vitamin D3 could alleviate lung fibrosis in mice in mouse pneumoconiosis (99). In detail, the original research demonstrated that Vitamin D3 could regulate macrophage polarization, which is the crucial step of immune response in injured AECs. Firstly, CD68 and Mrc1 represent two different identities of the classical macrophage classification of M1 and M2 (100). Secondly, single-cell RNA-sequencing data suggested that the Mrc1 expression increased in alveolar macrophages after 9-month coal dust particle stimulations (101). The original study proved that vitamin D3 supplementation could inhibit the over-activated M2 through immune fluorescence staining and regulate the TGF-β pathway (102). As we know, the TGF-β pathway plays a critical role in lung inflammation and is a recognized indicator of fibrosis. To be summarized, Vitamin D exerts its beneficial effects on many macrophage components, such as modulating phagocytic activity and cytokine production. Significantly, Vitamin D could alleviate the inflammatory injury of macrophage polarization (103). Thereby, in future studies, vitamin D supplementation might be a clinical strategy to solve the disorder of lung fibrosis.

### 5.3 Exosomal miRNAs

Firstly, exosomes are cell-secreted, nanosized, bi-lipid vesicles continuously secreted from various cells. AECs, inflammatory cells, and fibroblasts could be the secreted cell (99–101). In the second place, exosomes contribute to biological processes by transporting various bioactive molecules, such as miRNAs, proteins, and lipids. These bioinformatic performers could execute different functions during the pathophysiological mechanism of IPF (104). For instance, the exosomes regulate angiogenic pathways by transferring miRNAs. Furtherly alleviate the collapse and destruction of pulmonary capillaries in IPF patients (105–107). Thirdly, exosomes could promote the polarization of macrophages when the lung microbiota changes. For example, M2 as the executor, responded to unrelenting lung injury. However, the overreaction of M2 under the influence of chemotactic factors could start the waterfall effect of the inflammatory storm. It indirectly accelerates the inflammatory exudation at the early stage of lung fibrosis. Oppositely, the exosome could hind the secretion of inflammatory chemotactic factors, including IL-13 and CCL-27 (67–69). Hence, future research focuses on the relationship between the immune regulation of IPF and microRNA secreted from the exosomes.

In 2021, an original Chinese study reported that exosomes could treat pulmonary fibrosis in mice *via* the therapeutic function of microRNA-29 (miR-29). And microRNA-29 could alleviate the occurrence of pulmonary fibrosis by downregulating the TGF-β/Smad3 signaling pathway in lung fibrosis mice (104). In this case, exosomal miRNAs started as the potential therapeutic option for end-stage pulmonary fibrosis. Firstly, miRNAs are a class of highly conserved endogenous small non-coding RNAs widely distributed in animals, which regulate cell differentiation, proliferation, and apoptosis by degrading the target mRNAs or inhibiting translation to regulate gene expression (105, 106). In other words, the miRNA plays a significant role in regulating the immunological function and protein expression (107). Secondly, evidence suggests that miRNAs are not randomly integrated into exosomes. MiRNAs are more prevalent in exosomes than in the cells from which they originate (108). Thirdly, introduce the function in detail. As we know, Alveolar epithelial cell type II (AEC-II) apoptosis is a critical determinant in the initiation and development of lung fibrosis (109). miRNA-30a is downregulated in a murine bleomycin-induced lung fibrosis model (110).

Furthermore, miRNA-30a overexpression has been proven to inhibit AEC-II apoptosis by dampening mitochondrial fission through dynamin-related protein (111). In contrast, TGF-β, as the classic pathogenic pathway, also could inhibit the expression of miRNA-29c and Fac receptors, causing AEC-II apoptosis and fibrosis (112). Fourthly, miRNA was closely related to inflammatory storms and autophagy (113). The inflammatory battery is associated with the onset of lung fibrosis. For instance, miRNA-506 was shown to target the 3’-UTR of nuclear factor-κB (NF-κB)/p65 to reduce its expression, and p65 Inhibition significantly reduced lung fibrosis and inflammation (114). Thus, miRNA-506 may regulate the inflammatory response in pulmonary fibrosis.

On the other hand, FOXO3a is one of the targets of miRNA-96; miRNA-96 expression is reduced in carbon black nanoparticle (CBNP) induced injured epithelial cells, which is accompanied by a significant increase in the expression of α-smooth muscle actin (α-SMA) (115). These effects were suppressed following miRNA-96 transfection, suggesting that miRNA-96 silencing can lead to an upregulation of FOXO3a, thereby stimulating pulmonary ECM. Nevertheless, miRNA-21 expression was significantly elevated in a nano-nickel-induced murine lung injury model, and fibrosis and miRNA-21 silencing inhibited TGF-β1 signaling and alleviated lung fibrosis (116). Regarding autophagy, autophagic cell death, also known as type-II programmed cell death, is a biological phenomenon that promotes eukaryotic cell regeneration (117). Previous studies suggested the downregulated expression of miRNA-326 in the fibrotic lung tissue of mice.

In contrast, miRNA-326 upregulation could alleviate silica-induced lung fibrosis *in vivo* research (118). In other words, the overexpression of miRNA-326 can inhibit silica-induced lung fibrosis by inhibiting inflammation and promoting autophagy by targeting TNFSF14 and PTBP1. Fifthly, miRNA also plays a significant role in proliferation and differentiation (119). In pulmonary fibrosis, fibroblasts differentiate into myofibroblasts, which may secrete a more incredible amount of ECM and collagen components than fibroblasts, aggravating lung fibrosis (120). For instance, the downregulated miRNA−7 expression is in polymyositis−associated interstitial lung diseases (PM-ILD) (121).

Meanwhile, another study revealed that miRNA−7 attenuated the proliferation and differentiation of fibroblasts by inhibiting SMAD2 expression (122). However, other miRNA molecules, such as miRNA−30, miRNA −101, and miRNA −344, can inhibit fibrosis by suppressing fibroblast proliferation, whereas miRNA−328 and miRNA −420 exert the opposite effect and stimulate the pathogenesis of lung fibrosis (123). Therefore, miRNA has the potential to apply to pulmonary fibrosis treatment. To be summarized, the miRNAs in exosomes come from a wide range of sources. Exosomes are nanoscopic particles that are still difficult to detect and isolate (124). Although the exosome isolation and identification techniques continuously evolve, there is a lack of standardization. Hence, reproducible methodology to quantify specific exosomes in clinical samples is still challenging.

### 5.4 3D Models of IPF and drug discovery

Despite significant research, effective therapies for IPF face challenges due to the lack of *in vitro* models to mimic disease pathophysiology. In this case, lung 3D cultures, including precision-cut lung slices (PCLS) and hydrogels, have emerged as valuable tools for drug discovery of IPF (125–127). For instance, a new study demonstrated PCLS maintains the native lung microbiota and is a relevant *in vitro* model to study lung fibrosis and drug testing in a diseased tissue condition (128). We hope that more prospective studies will be conducted to evaluate the efficacy and safety of PCLS in treating IPF. On the other hand, hydrogels are water-swollen crosslinked networks of polymers and offer another *in vitro* model to study IPF. Hydrogels can be customized to model normal or diseased microbiotas by altering biomaterials and crosslinking mechanisms (129). Although lung 3D technology is still in phase 1 of the clinical trial, more original research might open potential therapeutic options for IPF.

## 6 Highlight and limitation

The highlights in this article included three points as follows. Firstly, our manuscript is the first review to clarify the relationship between pulmonary microbiota and immune regulation. Secondly, we revised the difference in lung microbiota in different states in ILD. We also reviewed that core fucosylation is critical in immune regulation during lung microbiota disorder. Thirdly, we summarized potential targeted therapy based on the relationship between pulmonary microbiota and immune regulation. However, in terms of possible treatment, microRNA is still a challenge in the clinical application of lung fibrosis because there is no standardized method to acquire microRNA. In patients with pulmonary fibrosis, there is no dose-response study of vitamin D supplementation regulating pulmonary microbiota. In future studies, further research to investigate the potential treatments based on the precise relationship between lung microbiota and immune regulation. To better clarify the prospects for their future clinical application in this field.

## Author contributions

TZ conceived, performed the manuscript draft, and drew all the figures. Secondly, WS and LG corrected the manuscript. Finally, MZ and LY validated the final manuscript. All authors contributed to the article and approved the submitted version.
